# Case report: Spontaneous remission of synchronous endometrial and ovarian cancers following pregnancy

**DOI:** 10.3389/fonc.2022.1001677

**Published:** 2022-11-29

**Authors:** Diandra Daley, Malcolm Padwick, Sabina Mistry, Vivek Malhotra, Radhika Selvi Vikram, Paul Stanciu

**Affiliations:** ^1^ Gynaecology Department, West Hertfordshire Teaching Hospitals National Health Service (NHS) Trust, Watford, United Kingdom; ^2^ Pathology Department , West Hertfordshire Teaching Hospitals National Health Service (NHS) Trust, Watford, United Kingdom; ^3^ Radiology Department, West Hertfordshire Teaching Hospitals National Health Service (NHS) Trust, Watford, United Kingdom

**Keywords:** endometrial, endometrioid, ovarian, pregnancy, remission, synchronous

## Abstract

**Introduction:**

Endometrial cancer is one of the most common malignancies affecting women. It is uncommonly diagnosed in young women, particularly in the absence of abnormal vaginal bleeding symptoms.

**Case presentation:**

A 28-year-old woman was referred to our department with lower abdominal pain. Transvaginal ultrasound showed a complex right adnexal mass with mixed echogenicity. Magnetic resonance imaging (MRI) identified a right-sided, torted, cystic solid ovarian mass, and a polypoid lesion in the uterine cavity.

**Diagnosis:**

Following multidisciplinary team advice, hysteroscopic transcervical resection of endometrial polypoid mass and laparoscopic right salpingo-oophorectomy was performed. Histopathologic assessment of the endometrial tissue showed changes consistent with grade 1 endometrioid endometrial carcinoma, while the right ovarian mass showed a well-differentiated endometrioid carcinoma.

**Intervention:**

The patient underwent hormonal treatment and surveillance whilst making a final decision regarding further surgical management. However, the patient stopped hormonal treatment after 2 weeks, went abroad and absconded from treatment for 8 months. On her return, she had conceived naturally and was in the first trimester of pregnancy. Further management was postponed until the patient was 7 weeks postnatal. The patient was not keen on any further surgical management and opted for close surveillance with ultrasound scans and hysteroscopies with endometrial biopsies. All subsequent endometrial biopsies showed normal endometrium, with no evidence of hyperplasia or malignancy.

**Outcome:**

To date, the patient remains on a 6-monthly surveillance plan and is considered to have had complete natural remission of her endometrial and ovarian cancers following pregnancy.

**Conclusion:**

This unique case demonstrates a natural phenomenon, in which the complete, natural remission of endometrial and ovarian cancers occurred following pregnancy and childbirth. The aetiology may be related to the high progesterone levels occurring in pregnancy.

## Introduction

Endometrial cancer is one of the most common malignancies affecting women, with around 9,700 new cases diagnosed in the UK every year (1). Whilst the exact prevalence of endometrial cancer in young women is equivocal, it has been estimated that up to 14% of cases occur in young women aged less than 40 years (2–4). Risk factors associated with the incidence of endometrial cancer in young women include higher body mass index, nulliparity and polycystic ovarian syndrome (3, 5, 6).

We present the unique case of a young woman diagnosed with both early endometrial and ovarian cancer that eluded full surgical treatment and developed complete spontaneous remission following pregnancy and childbirth.

## Case presentation

### Case description

A 28-year-old female patient of Indian ethnicity was referred to our specialist gynaecology clinic after presenting to her general practitioner with a few weeks’ history of right-sided lower abdominal pain, radiating to the right flank. An ultrasound scan performed in primary care showed a right adnexal solid cystic mass, initiating urgent referral to the rapid access gynaecological cancer service.

The patient had no history of any medical conditions. She was nulliparous with a BMI of 27 and no previous gynaecological or surgical history. She described an unremarkable menstrual history with regular cycles, and no menorrhagia, dysmenorrhoea or intermenstrual bleeding. She was not using any regular medication or contraceptives, and had no family history of gynaecological conditions or cancer. Her 3-yearly cervical cytology testing was up to date and unremarkable. She was a non-smoker and consumed alcohol socially.

### Clinical findings

**Figure 1:**
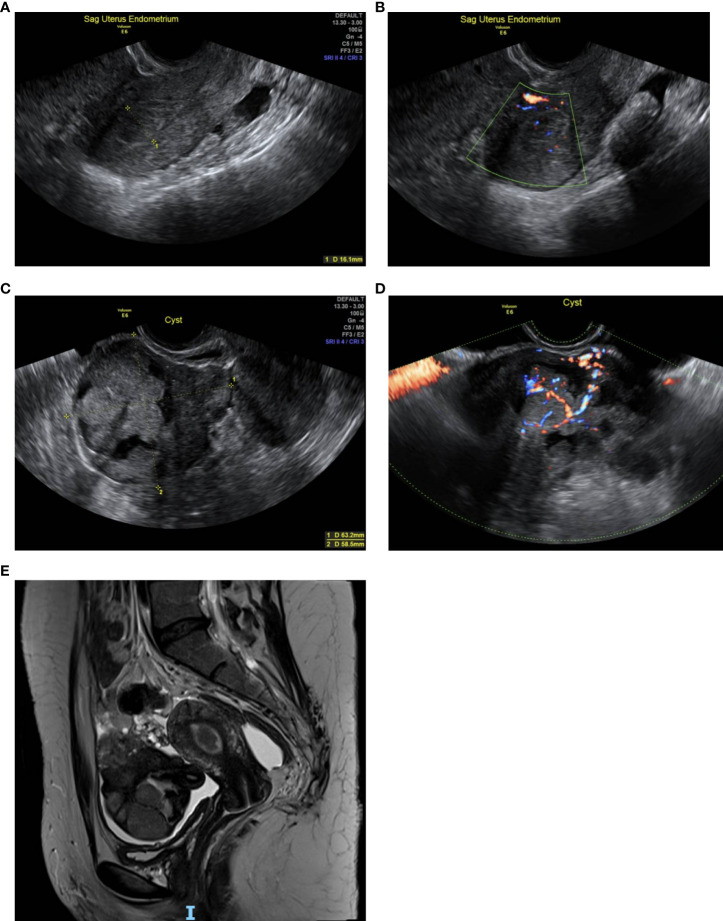
**(A)**: Transvaginal scan (TVS) of the uterus shows an endometrial thickness of 16 mm. **(B)**: TVS of the uterus shows anechoic areas with Doppler colour flow within the endometrial cavity. **(C)**: TVS shows a right adnexal solid cystic mass measuring 74 x 58 x 63 mm, with the solid component measuring 40 mm. **(D)**: TVS with colour Doppler, demonstrating moderate blood flow through the right adnexal mass. **(E)**: Pelvic MRI shows a polypoidal lesion in the uterine cavity. The right adnexal mass is seen within a pool of free ascitic fluid extending both behind and in front of the uterus.

Abdominal and bimanual vaginal examination was deemed inconclusive but revealed no obvious abnormalities. Vaginal speculum examination demonstrated no abnormalities of the vulva, vagina and cervix. A transvaginal ultrasound performed in the expert, specialist clinic showed an anteverted, normal sized uterus, with an endometrial thickness of 16mm. Anechoic areas with Doppler colour flow were seen in the endometrial cavity, suggestive of endometrial polyps. The rest of the endometrium was regular and well defined with preservation of the endo-myometrial junction. (**Figures 1A, B**). The left ovary appeared normal. In the right adnexa, a solid cystic mass measuring 74 x 58 x 63 mm was seen, with the solid component measuring 40 mm with shadowing (**Figure 1C**). The mass had a colour score of 3, suggestive of moderate blood flow through the mass (**Figure 1D**).

According to IOTA simple rules, the mass was indeterminate because of shadowing, a benign feature, and colour score of 3, which was a malignant feature (7).

The IOTA-ADNEX risk model was subsequently used to evaluate the ovarian lesion. This suggested a 44.2% risk of being benign and a 55.8% risk of ovarian malignancy, of which borderline was 36.7%, followed by a 12% risk of being stage 1 ovarian cancer. Risk of stage 2-4 ovarian cancer was 5.2%. Risk of metastatic ovarian cancer was 2% (8).

On subjective assessment, the mass was suspected to be a sex-cord stromal tumour.

### Diagnostic assessment

Following initial assessment, due to the indeterminate nature of the adnexal mass, a plan was made to obtain serum tumour markers, perform magnetic resonance imaging (MRI) and to discuss the case in the specialist gynaecological cancer multidisciplinary team (MDT) meeting.

All tumour markers tested were normal (CA125 29, AFP 1, HCG <2, and LDH 153).

Pelvic MRI showed a polypoid lesion in the uterine cavity. The left ovary appeared normal. The right adnexal mass was identified within a pool of free ascitic fluid, which extended both behind and in front of the uterus. The mass had bright uptake on T1 weighted series and featured small cysts, suggestive of an enlarged, torted, solid cystic right ovarian mass (**Figure 1E**). The MRI was inconclusive of malignancy because of the distorted architecture caused by the torsion. A staging computer tomography (CT) of the chest, abdomen and pelvis did not show any evidence of local or distant metastasis.

Hysteroscopy unveiled multiple polyps in the lower uterine cavity; the endometrium appeared normal at the uterine fundus. Transcervical resection of the uterine polyps was performed. Laparoscopy confirmed an 8cm right-sided, cystic and solid, torted mass with omental and peritoneal adhesions and minimal inflammatory ascites. Following removal of the ascitic fluid, careful examination did not reveal any other abnormalities inside the abdominal cavity. Adhesiolysis followed by uncomplicated right salpingo-oophorectomy was performed and the specimen was removed inside an Endobag without spillage. All the specimens were sent for histological analysis.

**Figure 2:**
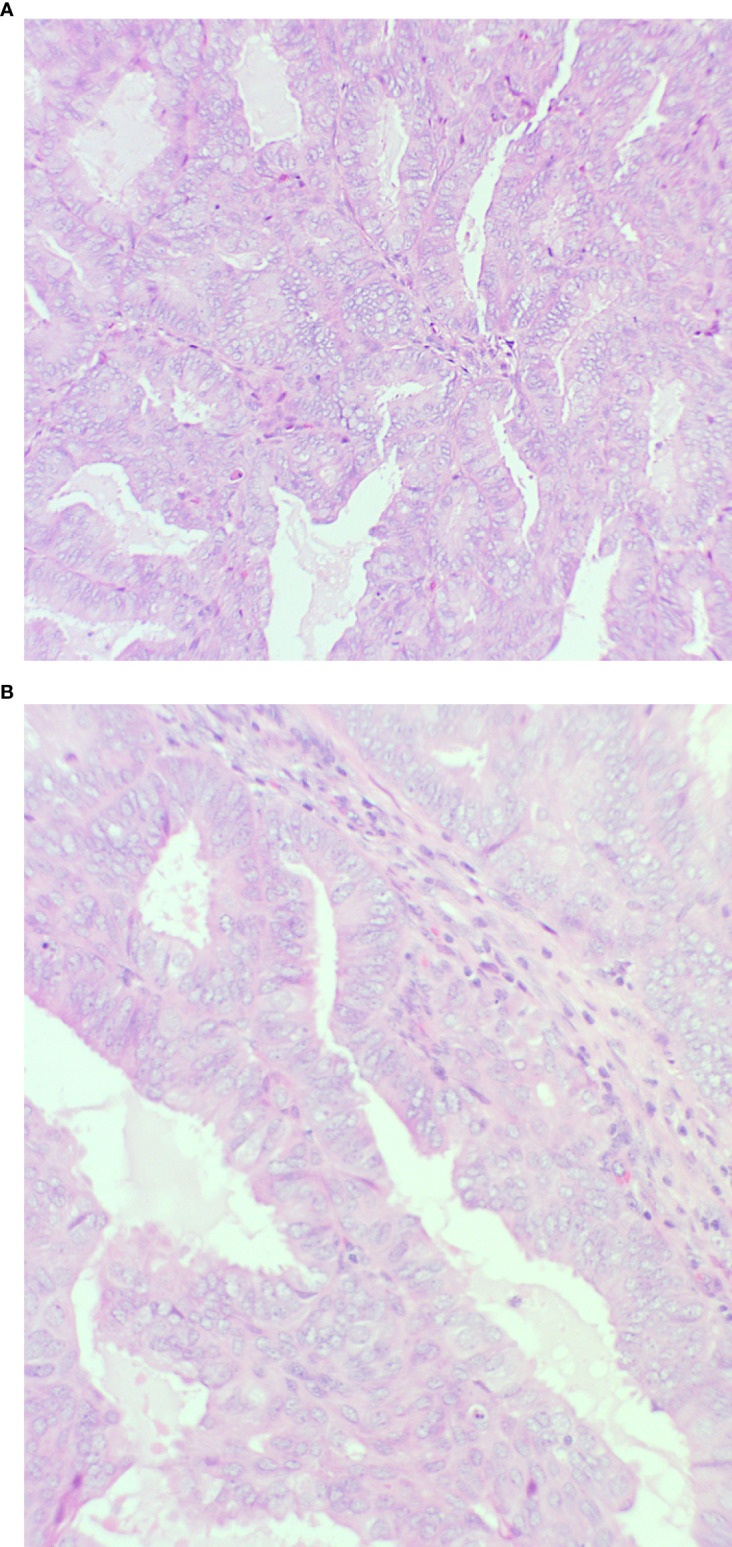
Endometrium showing features of an endometrioid endometrial carcinoma **(A)**: x20 magnification and **(B)**: x40 magnification. There is a glandular proliferation with no intervening stroma composed of cells with pseudostratified nuclei. Mild nuclear atypia is present consistent with grade 1.

**Figure 3:**
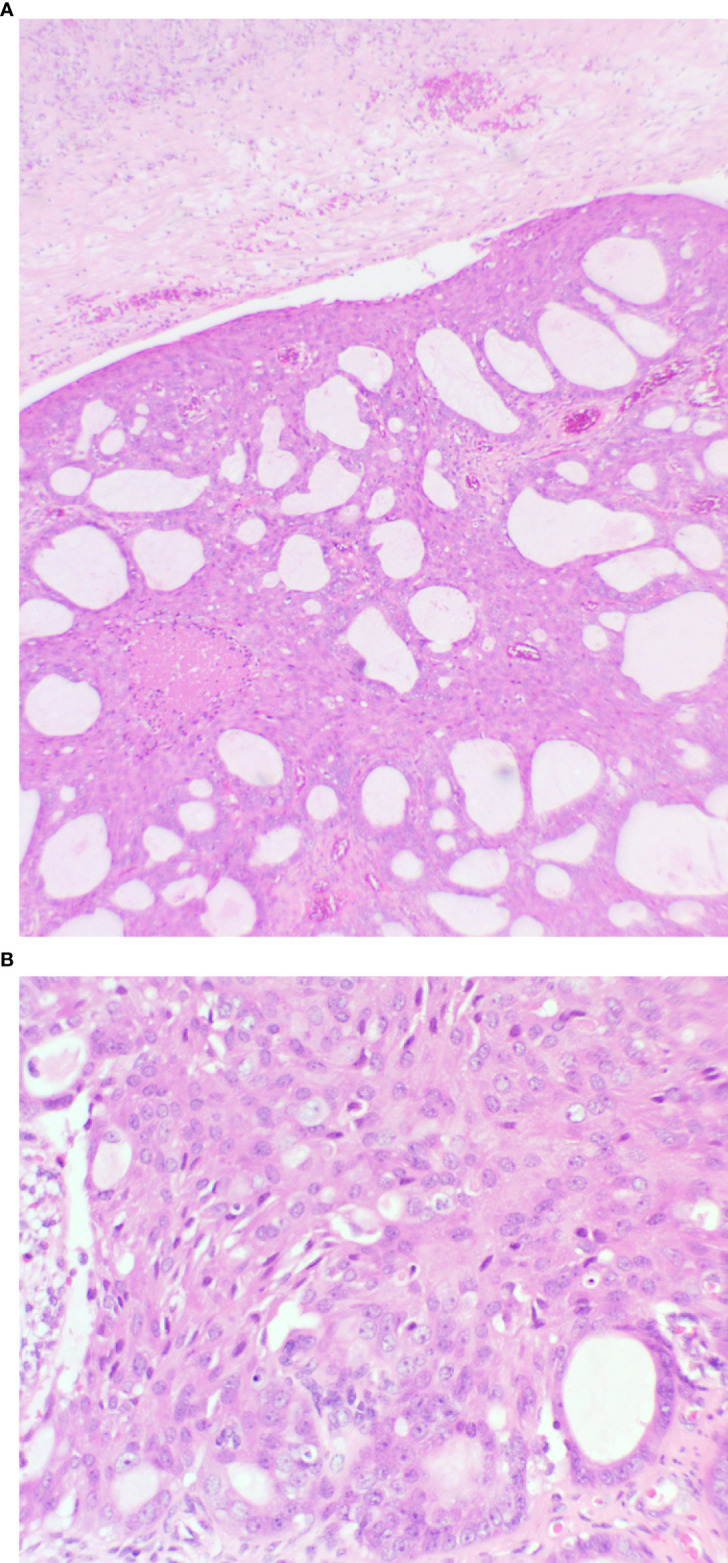
Ovary showing replacement by variably sized glands **(A)**: x20 magnification, and solid areas **(B)**: x40 magnification. The glands are lined by endometrioid type epithelium with cytological atypia. Necrosis is present.

The endometrial polypoid tissue showed changes consistent with grade 1 endometrioid endometrial adenocarcinoma (**Figures 2A, B**). Sections from the right ovarian mass showed features in keeping with a well-differentiated endometrioid ovarian carcinoma (**Figures 3A, B**). Possible endometriosis was also identified. It was uncertain as to whether the endometrial and ovarian lesions were synchronous tumours or represented metastatic deposits. Immunohistochemistry for mismatch repair proteins (MMR) was undertaken on the right ovarian mass and the tumour cells showed normal nuclear staining for MLH1, PMS2, MSH2 and MSH6.

The patient was re-discussed at the specialist oncology MDT meeting. Based on the histological findings, the MDT consensus was of a diagnosis of International Federation of Gynaecology and Obstetrics (FIGO) stage 1A, grade 1 endometrioid endometrial adenocarcinoma, and FIGO stage 1A well differentiated endometrioid carcinoma of the right ovary, incompletely staged.

## Therapeutic intervention

The consensus recommendation was that the patient should be offered completion surgery, including total abdominal hysterectomy, left salpingo-oophorectomy, with omentectomy and lymphadenectomy. In light of the patient’s young age and nulliparity, she was also offered an alternative, fertility-sparing, hormonal treatment option with oral Megestrol acetate 160mg daily, and surveillance with subsequent hysteroscopy and laparoscopy 6-8 weeks later.

The patient was undecided on the type of therapeutic intervention she wished to receive and was due to travel abroad soon after being informed of her diagnosis. She was therefore prescribed the hormonal treatment and prophylactic enoxaparin sodium, with a plan to re-discuss definitive treatment on her return to the UK.

## Outcome and follow-up

The patient remained abroad and absconded from treatment. After 2 months of unsuccessful attempts to make contact with the patient, she was subsequently discharged back to primary care. Eight months later, the patient re-presented to her GP and was urgently referred back to gynaecology clinic. On assessment, the patient reported that she had not received any medical treatments or alternative therapies in the previous eight months whilst abroad, and had stopped taking Megestrol acetate after 2 weeks due to intolerable side effects. The patient had conceived naturally and was 9 weeks pregnant at the time of assessment.

In line with the patient’s wishes, an MDT decision was made to postpone further investigation and treatment until the patient had delivered her baby, to avoid compromising her pregnancy. She declined VTE prophylaxis throughout her pregnancy.

Her pregnancy was followed up by the local high-risk obstetric team. All antenatal ultrasound scans were unremarkable with no abnormalities detected. The patient had an uncomplicated pregnancy and had a spontaneous vaginal delivery at term. Histological analysis of the placenta showed features of chorioamnionitis only.

The patient was re-assessed in outpatient gynaecology clinic at 7 weeks postnatal. She was amenorrhoeic and breastfeeding. Speculum examination demonstrated normal vulva, vagina and cervix. Transvaginal ultrasound was unremarkable, with an endometrial thickness of 3.9mm, and a normal appearing left ovary. An endometrial biopsy obtained by pipelle showed hyalinised decidua and inactive endometrium, with no evidence of hyperplasia or neoplasia. Repeat tumour markers including CA125 were normal.

Repeat hysteroscopy was unremarkable with no new polyps seen. Endometrial biopsy taken intra-operatively showed proliferative changes with no evidence of hyperplasia or malignancy. The gynaecological oncology MDT recommendation was for surveillance with repeat ultrasound, hysteroscopy and tumour markers at 8 months postnatal.

At 8 months postnatal the patient’s menstrual periods had recommenced, with no menstrual irregularities or intermenstrual bleeding. Repeat ultrasound showed a normal uterus with regular echotexture and an endometrial thickness of 8mm. The left ovary remained normal in appearance. Hysteroscopy was postponed on the basis of reassuring ultrasound findings.

The patient was reviewed again at 14 months postnatal. Repeat ultrasound showed a regular, homogenous endometrium with a thickness of 11mm. The left ovary appeared normal. Tumour markers were repeated and remained normal. Hysteroscopy revealed a normal appearing endometrium with no polyps. An endometrial pipelle biopsy showed a late proliferative endometrium with no hyperplasia or invasive malignancy.

Repeat diagnostic laparoscopy was considered to evaluate the left adnexa as part of ongoing surveillance. However, the patient wished to avoid further surgery unless indicated from other procedure findings.

To date, the patient remains on a 6-monthly surveillance plan consisting of pelvic ultrasound and tumour markers as routine. Indication for hysteroscopy will be evaluated during assessment. Laparoscopy will be recommended in instances of abnormal or inconclusive findings. The patient is considered to have had complete natural remission of her endometrial cancer following pregnancy.

Completion surgery, including a contralateral salpingo-oopherectomy and total abdominal hysterectomy, will be offered to the patient once her family is complete.

**Table 1** summarises the relevant historical and current data from the episode of care.

**Table 1:** Timeline of relevant historical and current data from the episode of care.

Dates	Relevant Past Medical History and Interventions			
**October-November 2018**	28-year-old nulliparous female Indian ethnicity BMI 24 Regular menstrual cycles with no dysfunctional uterine bleeding No previous gynaecological, medical or surgical history		No regular medication or contraceptives Cervical cytology testing up to date and unremarkable Non-smoker; consumed alcohol socially No family history of gynaecological conditions or cancer	
**Date**	**Summaries from Initial and Follow-up Visits**	**Diagnostic Testing** **(including dates)**		**Interventions**
November 2018 (initial visit)	Presenting complaint: right-sided lower abdominal pain radiating to right flank Abdominal and bimanual vaginal examination: inconclusive Vaginal speculum examination: NAD	TV US (16/11/18): right adnexal mass with mixed echogenicity		19/12/18: Hysteroscopy, transcervical resection of uterine polyp, laparoscopic right salpingo-oophorectomy and adhesiolysis
		TV US (27/11/18): Left ovary normal; ET 16mm. Right adnexa: 74mm cystic solid adnexal mass with Doppler flow seen		
		Tumour markers 27/11/18: normal (CA125 29, AFP 1, HCG <2, LDH 153)		
		- MRI Pelvis (06/12/2018): Polypoidal lesion in uterine cavity; left ovary – NAD; an enlarged, torted, cystic solid right ovarian mass with free ascitic fluid		
		Histology (20/12/2018): - Endometrium: Grade 1 endometrioid endometrial carcinoma - Right ovary: well-differentiated endometrioid carcinoma - Immunohistochemistry: normal expression of MMR proteins		
		CT chest, abdomen and pelvis (02/01/19): no metastases		
January 2019 (follow up)	MDT outcome: Recommendation for completion surgery OR oral megestrol acetate with 6-8 weekly surveillance Patient undecided about surgery; prescribed megestrol acetate to start whilst abroad; plan to review in 4-6 weeks Patient remained abroad, absconded from treatment. Discharged from gynaecology secondary care	Nil		03/01/19: oral Megestrol acetate 160mg daily – stopped taking after 2 weeks No interventions received whilst abroad
September 2019 (follow up)	Patient returned to the UK and re-referred to gynaecology secondary care Patient 9-10 weeks pregnant on re-assessment MDT outcome: postpone further investigation and treatment until delivery	Nil		Nil
June 2020 (follow up)	Seen in clinic at 7 weeks postnatal Amenorrhoeic and breastfeeding No new/recurrent symptoms Vaginal speculum examination: NAD	TV US (30/06/20): unremarkable, ET 3.9mm, left ovary - NAD		Hysteroscopy (20/07/20): NAD
		Histology (30/06/20): - Endometrial (pipelle) biopsy: no evidence of hyperplasia or neoplasia	
		Tumour markers (30/06/2020): normal	
		Histology (20/07/20: - Endometrium: no evidence of hyperplasia or malignancy	
February 2021 (follow up)	8 months postnatal Return of normal menstrual cycels; no dysfunctional uterine bleeding No new/recurrent symptoms	TV US (14/01/21): normal uterus and left ovary; ET 8mm		Hysteroscopy postponed as normal ultrasound
August 2021 (follow up)	14 months postnatal No new/recurrent symptoms	TV US (31/08/21): normal uterus and left ovary; ET 11mm		Hysteroscopy (25/10/21): NAD
		Tumour markers (31/08/2021): normal	
		Histology (25/10/21): - Endometrial (pipelle) biopsy: no evidence of hyperplasia or malignancy.	
October 2021- present	Follow up imaging negative to date, observing with 6-monthly surveillance			

## Discussion

Spontaneous regression has been acknowledged as a natural phenomenon in relation to many types of cancer, and has been discussed in medical literature as early as 1742 (9). More recently, a review of previous reports of tumour regression has identified a common association between spontaneous regression of cancers and acute infection, fever, and subsequent activation of the immune response (10). Whilst there are a small number of cases in which spontaneous regression of endometrial cancers has been observed, they describe cases of postmenopausal women, or those with advanced, distant metastatic disease (11–14). However, the spontaneous regression of endometrial cancer following pregnancy, in the absence of any comprehensive intervention, is extremely rare. A literature review failed to identify cases where the patient was diagnosed with endometrial cancer before conceiving, and whom their endometrial cancer resolved following pregnancy, without any substantial hormonal or surgical treatment.

The particularity of this case is that, apart from nulliparity, the patient lacked any recognisable risk factors for endometrial cancer, such as exposure to unopposed oestrogen, obesity, diabetes, polycystic ovarian syndrome, and use of tamoxifen (15). Whilst this patient’s age at menarche is unknown, endometrial cancer risk amongst nulliparous women is not thought to be associated with age at menarche (16). However, this patient was found to have multiple endometrial polyps. It is unclear how long she had uterine polyps before their detection, and whether this influenced their malignant transformation. Whilst endometrial cancer occurs in only 2-5% of women with endometrial polyps (17), the malignant potential of polyps in this case remains unclear.

The atypical occurrence of endometrial cancer in the absence of dysfunctional uterine bleeding must also be noted. Very few studies describe cases of endometrial cancer without abnormal bleeding, and such cases are exclusive to postmenopausal women (18, 19).

Various hypotheses for the protective effect of pregnancy on endometrial malignancy have been discussed. The persistently high progesterone levels occurring in pregnancy are thought to arrest mitotic activity and suppress carcinogenesis during this period (20). This may explain the remission observed in this case, and concurs with the rationale of progestin use in fertility-sparing treatment of endometrial cancers of the same grade. Others have proposed a physical “clearance” of premalignant and malignant cells or lesions during delivery (21), or tissue remodelling during postpartum involution of the uterus that limits growth of malignant tumours (22).

Synchronous endometrial and ovarian carcinomas are a rare occurrence, but accounts for the majority of synchronous female genital tract tumour combinations (23). Approximately 50-70% of these cases demonstrate endometrial and ovarian carcinomas both of endometrioid type, making a diagnosis of synchronous cancers difficult (23). Criteria for distinguishing synchronous tumours from metastatic deposits was first detailed by Ulbright and Roth (24), and further developed by Scully et al. (25). The histological findings in this case meet various minor criteria for synchronous tumours: ovarian tumour unilaterality; absence of lymphovascular emboli; no or only superficial myometrial invasion of endometrial tumour; and absence of evidence of distant spread (25, 26). Although it is uncertain, the endometrial and ovarian lesions diagnosed in this case could be classified as synchronous tumours on the basis that at least two criterion have been met.

There is also evidence to suggest increased rates of synchronous tumours in young women with endometrial cancer. A recent retrospective study identified a significantly higher rate of synchronous ovarian cancer in young women aged ≤40 years with endometrial cancer, compared to those aged 41-60 years (9.2% vs 0.7%, *P*<0.001) (5). This rate has previously been suggested to be as high as 19% in women aged less than 50 years (6). Moreover, synchronous endometrial and ovarian cancers generally have a better prognosis when compared to metastatic lesions (27, 28). The favourable outcome observed in this case further promotes the likelihood of synchronous cancers.

At present, surgical treatment for this patient’s ovarian and endometrial cancer is considered incomplete. However, the indication for further surgery without evidence of recurrence could be debated. Findings of a recent meta-analysis suggested that hysterectomy is associated with a lower risk of recurrence for FIGO stage I ovarian tumours (OR 0.23, *P=*0.0006), but an increased risk of death (due to disease or of any other cause) when compared to uterine-sparing surgery for borderline ovarian tumours (29). Without recurrence of this patient’s ovarian cancer, the risks associated with hysterectomy - besides loss of fertility - will need to be closely examined as part of future planning.

Contrastingly, the recurrence of FIGO stage I ovarian cancer during pregnancy and following salpingo-oopherectomy has been observed (30). Considering the clinical course in such cases, it has been suggested that minimal interaction occurs between the intrauterine and intrabdominal environments, and pregnancy may in fact initiate or accelerate recurrence. Whilst remission of ovarian cancer has occurred in this case, the risk of future recurrence is therefore unclear.

Based on The Cancer Genome Atlas 2013 molecular analysis of endometrial cancers, the carcinoma identified in this case can be categorised under the copy-number low/endometrioid subtype (31, 32). Analogous to this classification, the patient’s endometrial cancer is considered to be of low prognostic risk under the most recent European Society of Gynaecological Oncology (ESGO), the European SocieTy for Radiotherapy and Oncology (ESTRO), and the European Society of Pathology (ESP) guidelines (33). Examining such molecular prognostic factors is particularly valuable in this instance, where atypical clinical factors may cause uncertainty regarding appropriate management.

Where the intrauterine conditions produced by pregnancy may equate to those occurring during progestin therapy, such molecular-level prognostic factors may also be applied to stratify the efficacy and favourability of fertility-sparing endometrial cancer treatment in the future.

The management of this case was strengthened by the comprehensive approach taken towards the patient’s diagnosis, taking into consideration important personal factors such as her age and her fertility wishes. In light of this, we devised a personalised treatment plan that provided the patient a choice of either fertility sparing conservative or radical surgical treatment.

A limitation of this case is the period during which the patient temporarily absconded from treatment. During this 8-month period, the patient did not have any repeat imaging – or any other form of disease surveillance. We are therefore unable to map out the exact course of her remission, and understanding of the exact disease process in this case remains unclear.

This unique case demonstrates a natural phenomenon, and highlights the potential for pregnancy to induce remission of gynaecological cancers. Whilst endometrial cancer is uncommon in young patients without abnormal bleeding, it is exceptionally rare to observe the complete, natural remission of endometrial cancer following pregnancy and childbirth. Natural remission of ovarian cancer under these circumstances is similarly extraordinary. In such cases, the aetiology may be related to the high progesterone-producing state of pregnancy. Further research is required to better understand the mechanism by which endometrial carcinogenesis may be arrested or reversed under the specific intrauterine conditions of pregnancy. How such conditions may affect extra-uterine gynaecological tumours must also be understood. In turn, the modification of current progestin therapy regimens - to achieve pregnancy levels of progesterone - may further enhance the prognostic benefit of this fertility-sparing treatment option for low-grade endometrial cancers. Such benefit may also be applicable to borderline or early-stage ovarian cancers in the future.

## Patient perspective

The patient declined to formally comment on her perspective on this episode of care.

## Data availability statement

The original contributions presented in the study are included in the article/Supplementary Material. Further inquiries can be directed to the corresponding author.

## Ethics statement

Ethical review and approval was not required for the study on human participants in accordance with the local legislation and institutional requirements. The patients/participants provided their written informed consent to participate in this study. Written informed consent was obtained from the individual(s) for the publication of any potentially identifiable images or data included in this article.

## Author contributions

Acquisition of data: DD, RV, PS, SM, and VM. Manuscript writing: DD, PS, RV, and SM. Critical review of the manuscript: All authors. All authors contributed to the article and approved the submitted version.

## Conflict of interest

The authors declare that the research was conducted in the absence of any commercial or financial relationships that could be construed as a potential conflict of interest.

## Publisher’s note

All claims expressed in this article are solely those of the authors and do not necessarily represent those of their affiliated organizations, or those of the publisher, the editors and the reviewers. Any product that may be evaluated in this article, or claim that may be made by its manufacturer, is not guaranteed or endorsed by the publisher.
